# High serum uric acid is associated with increased arterial stiffness in hypertension

**DOI:** 10.18632/aging.103506

**Published:** 2020-07-23

**Authors:** Li-Na An, Ning Rong, Min Ning, Liu-Liu Feng, Zhen-Han Chen, Wei-Qing Liu, Xiao-Chun Ouyang, Fan-Rong Diao, Zhi-Gang Han, Jiang Hong

**Affiliations:** 1 Department of Geriatrics, Shanghai First People’s Hospital Affiliated to Nanjing Medical University, Shanghai 200080, China; 2 Department of Cardiology, Shidong Hospital of Shanghai Yangpu, Shanghai 200433, China; 3 Department of Neurology, School of Clinical Medicine, Dali University, YunNan 671000, China; 4 Community Health Service Center, Shanghai 200435, China; 5 Community Health Service Center, Shanghai 201914, China; 6 Department of Neurology, No.908 Hospital of the People's Liberation Army Joint Logistics Support Force, JiangXi 330000, China; 7 Department of Cardiology, Changhai Hospital, Naval Military Medical University, Shanghai 200433, China; 8 Department of Radiology, Obstetrics and Gynecology Hospital of Fudan University, Shanghai 200011, China; 9 Department of Emergency, Shanghai First People's Hospital Affiliated to Nanjing Medical University, Shanghai 200080, China

**Keywords:** arterial stiffness, pulse wave velocity, serum uric acid, hypertension, multivariate analysis

## Abstract

Serum uric acid level has been found to be associated with cerebrovascular diseases. However, whether serum uric acid level is a risk factor for arterial stiffness in the hypertension population is unclear. This study was designed to determine the relationship between serum uric acid level and arterial stiffness in the hypertension population. A total of 10450 participants were evaluated for the risk of arterial stiffness. Brachial-ankle pulse wave velocity (baPWV) was assessed, and high baPWV was determined as the highest quartile of baPWV values in a sex-specific manner. We evaluated the association between serum uric acid level and baPWV through multivariate-adjusted linear and logistic regression analyses. There was a significant difference on high baPWV between patients with quartiles of serum uric acid level in females and males (*p*<0.01), respectively. The odds ratios (95% CI) of the highest baPWV quartile across the sex-specific serum uric acid level were 1.0, 1.71 (1.35, 2.17), 1.75 (1.38, 2.23), and 1.95 (1.51, 2.51) in female, and 1.0, 1.33 (1.09, 1.64), 1.36 (1.11, 1.67), and 1.67 (1.36, 2.04) in male after adjusting for potential confounders. In conclusion, serum uric acid level could be considered as an important risk factor for arterial stiffness in Chinese hypertension population.

## INTRODUCTION

Higher serum uric acid is a potential independent risk factor for cardiovascular disease, stroke, metabolic syndrome and arteriosclerosis [1–3]. The Framingham Study, which was the first to evaluate the association between serum uric acid level and cardiovascular disease in general population, demonstrated that higher serum uric acid level was a risk factor of cardiovascular disease [4]. Additionally, a prospective cohort study has shown that hyperuricemia could be considered an important risk factor for the mortality from all causes, total cardiovascular disease, and ischemic stroke in general population [5].

Brachial-ankle pulse wave velocity (baPWV), widely applied to assess arteriosclerosis during health screening in a non-invasive manner, is an important indicator for arterial stiffness [6, 7]. Several previous studies have suggested that higher serum uric acid, which could increase the risk of arteriosclerosis, was associated with increased arterial stiffness estimated by baPWV in general population [8, 9].

However, previous epidemiologic and prospective studies on baPWV were only limited in healthy individuals and general population, and the value of high baPWV in the gender reported in those studies was controversial [10, 11]. In addition, higher serum uric acid level increased the risk for hypertension, which has a positive correlation with arterial stiffness [12–14]. Based on current evidence, the association of serum uric acid level and arterial stiffness remains unclear in the hypertension population and whether higher serum uric acid level is an important risk factor for arterial stiffness in the hypertension population needs to be further investigated. Thus, we conducted this cross-section study, aiming to assess the association between serum uric acid level and arterial stiffness in the hypertension population. To our knowledge, no such large-scale study has previously reported on the association between serum uric acid level and arterial stiffness in the hypertension population.

## RESULTS

### Baseline characteristics

**Figure 1:**
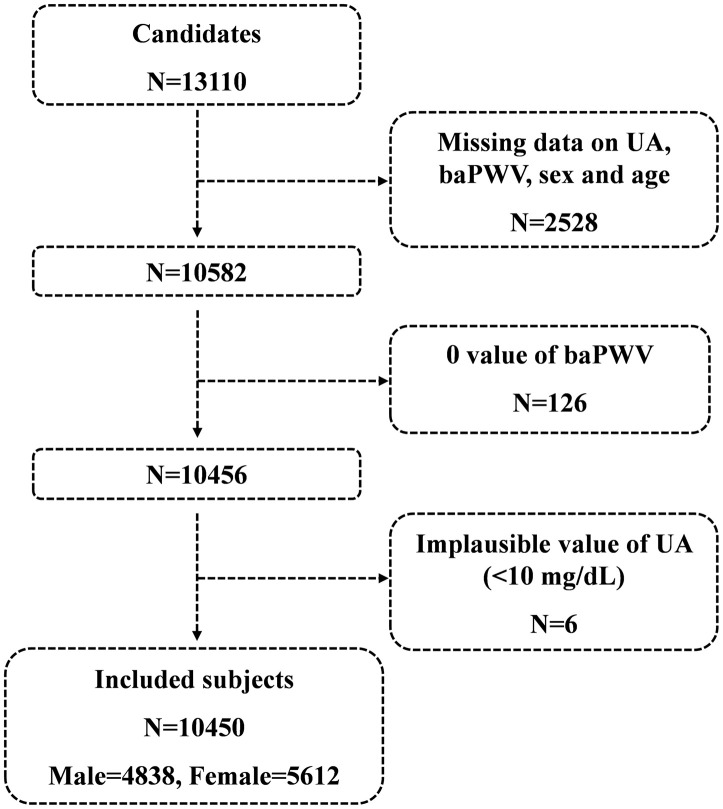
**Flowchart of the study.**

A total of 13110 candidates were recruited for the cross-section study at the time of the final survey in July 2017. Among these candidates, those who had missing data related to serum uric acid, baPWV, sex and age were excluded from the eligible candidates for this study (n=2528). Those with 0 value of baPWV (n=126) and those with implausible values of serum uric acid (<1mg/dl) (n=6) were also excluded from the pool of eligible candidates for this study. As a result, a total of 10450 subjects were included for the final analyses. A flowchart of the study is shown in Figure 1.

Among 10450 study subjects, males accounted for 46.30% (n=4838) and females for 53.70% (n=5612). The age of the enrolled subjects ranged from 44 to 83 yr (female, 44–83 yr; male, 45–83 yr) with a mean age of 64.71±7.45 yr (female, 63.77±7.38 yr; male, 65.79±7.38 yr). The mean serum uric acid level was found to be significantly lower in female (5.29±1.44 mg/dL) than in male (6.24±1.47 mg/dL, *p*<0.001). Therefore, sex-specific quartiles of serum uric acid were used. The median (range) serum uric acid values of 3.63 (1.55- 4.20), 4.72 (4.22- 5.11), 5.65 (5.13- 6.25) and 6.94 (6.27-12.55) mg/L were used for female, and 4.57 (2.29- 5.23), 5.63 (5.24- 6.17), 6.61 (6.18- 7.19) and 7.95 (7.21-11.76) mg/L were used for male (Table **1**).

The mean baPWV was significantly different among each serum uric acid quartile group in female and male, and the mean baPWV in the fourth quartile was more than that in the first quartile (both *p*<0.001). Among each serum uric acid quartile group, the body mass index (BMI), systolic blood pressure, diastolic blood pressure, heart rate, fasting glucose, total-cholesterol, high-density lipoprotein (HDL) cholesterol, and smoking status was significantly different in female (all *p*=0.672) but not significantly different in male (all *p*>0.001). The other baseline characteristics shown in Table 1 were significantly different among each serum uric acid quartile group in male and female participants (*p*<0.001 for all parameters).

**Table 1:** Baseline characteristics of participants by UA.

**Index**	**Q1**	**Q2**	**Q3**	**Q4**	***p* value**
**Female**					
Number	1387	1385	1418	1422	
UA median (min-max) (mg/dL)	3.63 (1.55-4.20)	4.72 (4.22-5.11)	5.65 (5.13-6.25)	6.94 (6.27-12.55)	<0.001
baPWV (cm/s)	1583.86±289.68	1662.40±329.18	1738.36±327.81	1770.38±361.16	<0.001
Age (years)	62.02±7.17	62.99±7.24	64.31±7.19	65.69±7.40	<0.001
BMI (kg/m^2^)	24.24±3.55	25.45±3.77	26.28±3.85	26.85±3.84	<0.001
SBP (mmHg)	137.09±12.79	139.78±13.38	141.21±13.55	143.22±15.45	<0.001
DBP (mmHg)	82.02±7.64	82.98±8.20	83.42±8.14	83.38±8.53	<0.001
Heart rate (bpm)	76.66±10.49	76.86±10.97	78.05±11.52	78.80±11.75	<0.001
**Dietary data (%)**					
Staple food (rice)	681 (49.42)	639 (46.47)	638 (45.15)	635 (44.91)	0.066
Vegetarian diet	688 (49.93)	708 (51.53)	716 (50.89)	722 (51.17)	0.855
**Laboratory data**					
GGT (U/L)	14.70 (12.10-19.20)	15.70 (12.50-20.22)	17.50 (14.00-23.80)	19.60 (14.90-26.50)	<0.001
Crea (μmol/L)	55.07±10.79	59.17±16.27	61.73±12.82	70.96±26.29	<0.001
Glu (mmol/L)	6.21±2.29	6.12±1.76	6.25±1.84	6.52±1.85	<0.001
**Serum lipid data**	5.18±1.03	5.38±1.02	5.48±1.11	5.68±1.20	<0.001
TCHO (mg/dL)	1.31±0.31	1.27±0.29	1.24±0.28	1.22±0.29	<0.001
HDL (mg/dL)	1.54±1.08	1.81±1.13	2.06±1.54	2.48±1.91	<0.001
TG (mg/dL)	1.29 (0.94-1.82)	1.52 (1.10-2.15)	1.73 (1.24-2.46)	2.00 (1.41-3.00)	<0.001
**Smoking (%)**					0.004
Never	1344 (97.53)	1321 (96.35)	1348 (95.47)	1340 (94.70)	
Former	10 (0.73)	17 (1.24)	28 (1.98)	33 (2.33)	
Current	24 (1.74)	33 (2.41)	36 (2.55)	42 (2.97)	
**Medication use (%)**					
Antihypertensive drugs	1237 (89.83)	1261 (91.98)	1329 (94.06)	1344 (95.39)	<0.001
Lipid lowering drugs	18 (1.31)	17 (1.25)	32 (2.28)	30 (2.14)	0.070
Antiplatelet drugs	20 (1.54)	34 (2.68)	36 (2.77)	39 (3.10)	0.063
**Male**					
Number	1201	1204	1223	1210	
UA median (min-max) (mg/dL)	4.57 (2.29-5.23)	5.63 (5.24-6.17)	6.61 (6.18-7.19)	7.95 (7.21-11.76)	<0.001
baPWV (cm/s)	1657.92±270.04	1709.63*±*197.65	1710.55*±*255.66	1731.16*±*352.50	<0.001
Age (years)	65.38*±*7.42	66.05*±*7.26	65.63*±*7.28	66.12*±*7.55	0.043
BMI (kg/m^2^)	23.77*±*3.58	23.99*±*3.54	24.09*±*3.61	24.09*±*3.66	0.100
SBP (mmHg)	139.24*±*11.48	139.79*±*13.02	140.45*±*12.41	140.42*±*13.67	0.073
DBP (mmHg)	83.88*±*8.39	83.82*±*8.60	84.14*±*8.29	84.01*±*8.59	0.809
Heart rate (bpm)	75.57*±*11.79	75.61*±*11.72	75.95*±*11.91	76.21*±*11.81	0.509
**Dietary data (%)**					
Staple food (rice)	566 (47.68)	552 (46.58)	594 (49.21)	617 (51.98)	0.050
Vegetarian diet	386 (32.60)	395 (33.36)	398 (33.11)	396 (33.36)	0.977
**Laboratory data**					
GGT (U/L)	21.75 (16.50-31.50)	23.00 (17.00-34.60)	23.00 (17.20-34.50)	24.60 (17.80-39.00)	<0.001
Crea (μmol/L)	75.78*±*14.61	79.55*±*22.98	81.63*±*32.87	85.47*±*47.18	<0.001
Glu (mmol/L)	6.19*±*1.87	6.15*±*1.76	6.17*±*1.91	6.17*±*1.74	0.981
**Serum lipid data**					
TCHO (mg/dL)	4.99*±*0.99	4.99*±*1.03	5.04*±*1.05	5.07*±*1.05	0.153
HDL (mg/dL)	1.28*±*0.33	1.28*±*0.34	1.29*±*0.34	1.27*±*0.33	0.546
TG (mg/dL)	1.14 (0.85-1.57)	1.16 (0.83-1.71)	1.24 (0.86-1.77)	1.26 (0.90-1.86)	<0.001
**Smoking (%)**					0.065
Never	290 (24.49)	293 (24.79)	306 (25.37)	321 (27.09)	
Former	283 (23.90)	310 (26.23)	290 (24.05)	327 (27.59)	
Current	611 (51.60)	579 (48.98)	610 (50.58)	537 (45.32)	
**Medication use (%)**					
Antihypertensive drugs	1107 (93.34)	1121 (94.76)	1134 (94.34)	1116 (94.74)	0.404
Lipid lowering drugs	9 (0.76)	14 (1.19)	21 (1.75)	13 (1.11)	0.167
Antiplatelet drugs	42 (3.69)	46 (4.15)	41 (3.60)	45 (4.03)	0.889

UA: Uric Acid, baPWV: Brachial-ankle pulse wave velocity, BMI: body mass index, SBP: systolic blood pressure, DBP: diastolic blood pressure, GGT: gamma-glutamyl transpeptidase, Crea: Creatinine, Glu: glucose, TCHO: total cholesterol, HDL: high-density lipoprotein, TG: triglycerides.

### Comparison of baPWV according to the UA quartile and gender

**Figure 2:**
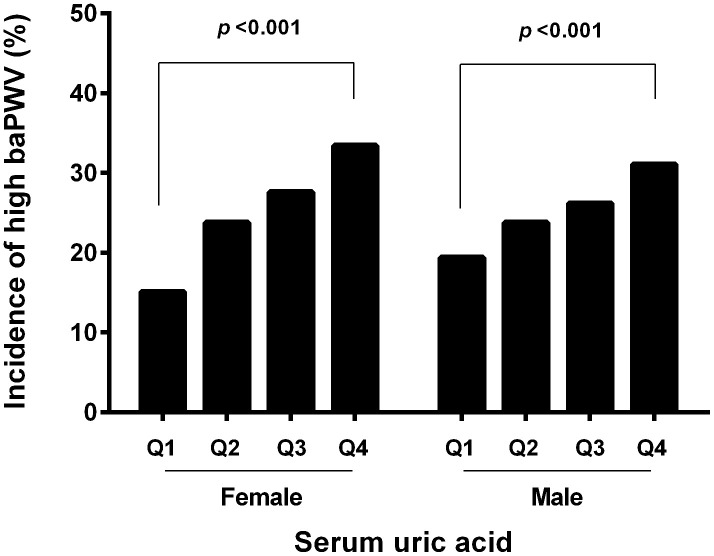
**Incidence of high baPWV according to the UA quartile and gender.** The incidence of high baPWV was increased with graded serum uric level in both genders.

The mean baPWV value was 1689.54±335.90 cm/s in female, which was significantly lower than that in male (1702.41±275.87, *p*=0.034). The sex-specific inter quartile cutoff points for baPWV were 1453, 1629, and 1876 cm/s in female, and those of 1519, 1681, 1851 cm/s in male. High baPWV was defined as the highest quartile of the values among the study subjects. Thus, the high baPWV was defined as equal to more than 1876 cm/s in female and 1851 cm/s in male. The incidence of high baPWV in the fourth quartile was more than that in the first quartile both in female (33.40% *vs* 15.07%, *p*<0.001) and in male (31.07% *vs* 19.40%, *p*<0.001). The prevalence of the high baPWV in the first, second, third and fourth serum uric acid quartiles in each gender is shown in Figure 2.

### Relationship between UA and baPWV

**Figure 3:**
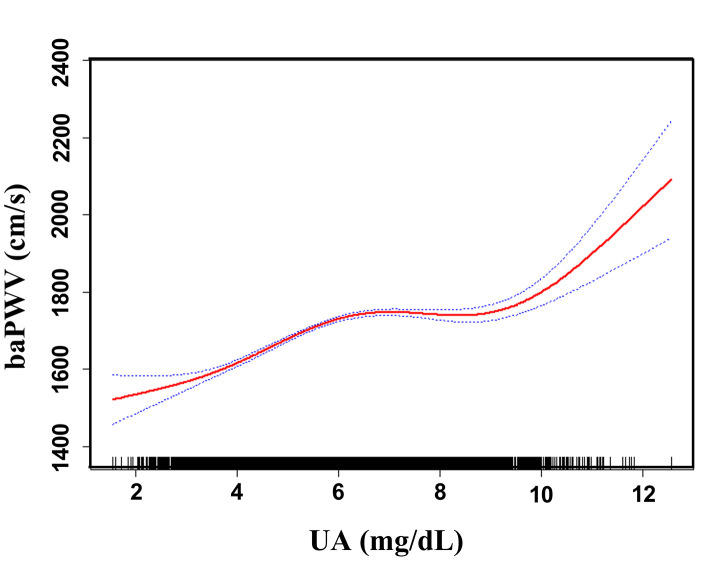
**Association between UA and baPWV.** A linear relationship between UA and baPWV was detected after adjusting for age, BMI, systolic blood pressure, diastolic blood pressure, heart rate, gamma-GTP, creatinine, fasting glucose, total cholesterol, HDL-cholesterol, triglycerides, smoking status, and antihypertensive drugs. Solid lines represent the fitting curve, and dotted lines represent the corresponding 95% CI.

**Figure 4:**
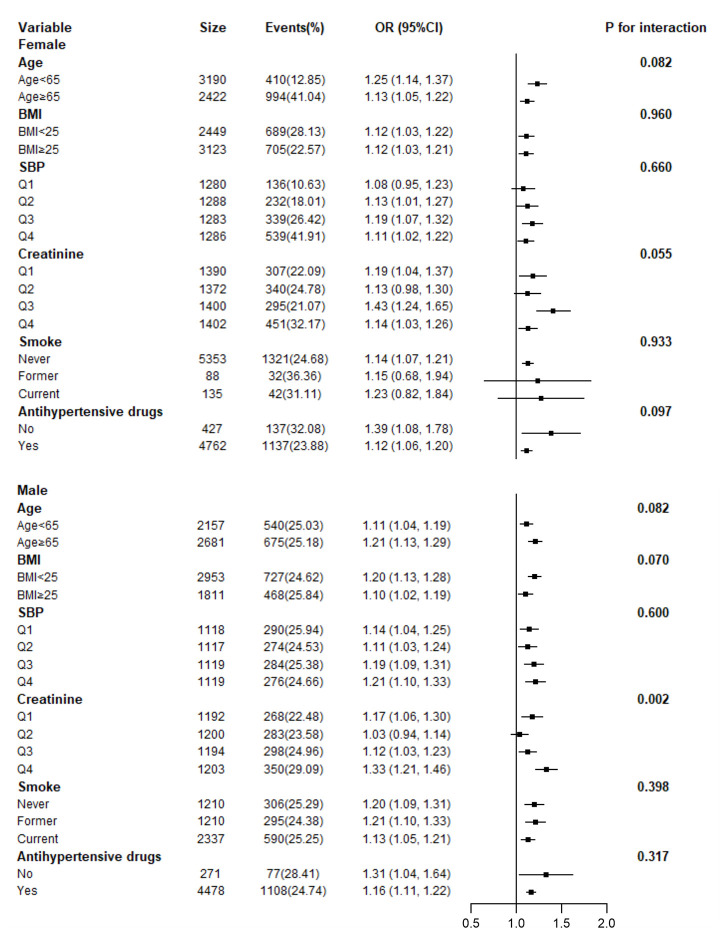
**Hierarchical analysis on relationship of UA and high baPWV.** Each stratification adjusted for all the factors (age, BMI, systolic blood pressure, diastolic blood pressure, heart rate, gamma-GTP, creatinine, fasting glucose, total cholesterol, HDL-cholesterol, triglycerides, smoking status, and antihypertensive drugs) except the stratification factor itself.

Adjusted smoothed plots suggest that there are linear relationships between the serum uric acid level and baPWV (Figure 3). Pearson’s correlation coefficients for the relationship between serum uric acid level and baPWV were 0.2048 (*p*<0.001) in female and 0.1284 (*p*=0.0001) in male. Scatter diagram between serum uric acid level and baPWV is shown in Supplementary Figure 1, and single factor logistic regression analysis of high baPWV is shown in Supplementary Table 1. Multiple logistic regression analysis without adjusting for confounder factors showed that the odds ratios (95%) for the relationship between serum uric acid and high baPWV were 1.27 (1.22-1.33, *p*<0.001) in female and 1.20 (1.15, 1.26, *p*<0.001) in male. When multivariate logistic analysis was performed after adjusting for age, BMI, systolic blood pressure, diastolic blood pressure, heart rate, gamma-glutamyl transpeptidase (GTP), creatinine, fasting glucose, total and HDL-cholesterol, triglycerides, smoking status, and antihypertensive drugs, the odds ratios (95%) for the relationship between serum uric acid and high baPWV were 1.13 (1.07- 1.21, *p*<0.001) in female and 1.16 (1.11- 1.22), *p*<0.001) in male, which showed that the association between serum uric acid level and high baPWV was statistically significant, and the odds ratios (95%) were 1.0, 1.71 (1.35, 2.17), 1.75 (1.38, 2.23), and 1.95 (1.51, 2.51) in female, and 1.0, 1.33 (1.09, 1.64), 1.36 (1.11, 1.67), and 1.67 (1.36, 2.04) in male after subdividing subjects according to the serum uric acid quartile and gender (Table 2.), which showed that the statistical significance was maintained. Hierarchical analysis, according to age, BMI, systolic blood pressure, triglycerides, smoking status, and antihypertensive drugs, also showed that the association between serum uric acid level and high baPWV was statistically significant (Figure 4, and Supplementary Table 2).

**Table 2:** Association between UA and high baPWV.

**Variable**	**Model 1**		**Model 2**	
	**Incidence, n (%)**	**OR (95%CI) *p***	**N**	**OR (95%CI) *p***
**Female**				
UA (continuous) 5612	1404 (25.02)	1.27 (1.22, 1.33) <0.001	4898	1.13 (1.07, 1.21) <0.001
log UA (categorical)				
Q1 (0.19-0.62) 1387	209 (15.07)	1		1
Q2 (0.63-0.71) 1385	329 (23.75)	1.76 (1.45, 2.13) <0.001		1.71 (1.35, 2.17) <0.001
Q3 (0.71-0.80) 1418	391 (27.57)	2.15 (1.78, 2.59) <0.001		1.75 (1.38, 2.23) <0.001
Q4 (0.80-1.10) 1422	475 (33.40)	2.83 (2.35, 3.40) <0.001		1.95 (1.51, 2.51) <0.001
**Male**				
UA (continuous) 4838	1215 (25.11)	1.20 (1.15, 1.26) <0.001	4248	1.16 (1.11, 1.22) <0.001
log UA (categorical)				
Q1(0.36-0.72) 1201	233 (19.40)	1		1
Q2 (0.72-0.79) 1208	286 (23.75)	1.29 (1.06, 1.57) 0.009		1.33 (1.09, 1.64) 0.006
Q3 (0.79-0.86) 1205	320 (26.17)	1.47 (1.22, 1.78) <0.001		1.36 (1.11, 1.67) 0.004
Q4 (0.86-1.07) 1215	376 (31.07)	1.87 (1.55, 2.26) <0.001		1.67 (1.36, 2.04) <0.001

Model 1: unadjusted

Model 2: adjusted for age, BMI, systolic blood pressure, diastolic blood pressure, heart rate, gamma-GTP, creatinine, fasting glucose, total cholesterol, HDL-cholesterol, triglycerides, smoking status, and antihypertensive drugs.

## DISCUSSION

This cross-section study showed that serum uric level was associated with arterial stiffness in the hypertension population. Our study found that a positive correlation between serum uric level and baPWV, and the incidence of high baPWV was increased with graded serum uric level in both genders. Furthermore, the association between serum uric level and arterial stiffness was independent after adjustment for other confounding atherogenic risk factors and hierarchical analysis. Therefore, these results demonstrated that higher serum uric acid level is a risk factor of arterial stiffness in the hypertension population.

In the present study, higher values of baPWV in both genders, including mean baPWV and high baPWV, were detected in our hypertension population than previous studies in general subjects. Our result showed that the mean baPWV values were 1689.54±335.90 cm/s in female and 1702.41±275.87 cm/s in male, which was higher than that of the Japanese study (1447±315 cm/s in female, n=297; 1549±342 cm/s in male, n=695), and the Korean study (1483.2±331.6 cm/s in female, n=3401; 1581.1±338.1 cm/s in male n=2167,) [8, 15]. High baPWV was defined as the highest quartile of the values among the study subjects. Therefore, the high baPWV values were 1876 cm/s in female and 1851 cm/s in male in our study, while the high baPWV values were 1594 cm/s in female and 1721 cm/s in male in the Japanese study [8]. Previous study has demonstrated that baPWV values over 1400 cm/s in female and 1600 cm/s in male could be a feasible cutoff point so as to assess aortic stiffness and predict cardiovascular mortality by the Framingham risk score [16]. Why higher baPWV values than before in gender were detected in our study? It may be explained by the main reason that participants in our study were hypertensives, while previous studies were performed in general subjects. It has been demonstrated that arterial stiffness was associated with hypertension, and arterial stiffness is increasingly recognized as an important index in patients with hypertension [17–19]. Therefore, it is reasonable to observe high baPWV in the hypertension population in our study. Besides, it is also related to the age of the enrolled subjects, ranged from 44 to 83 years, which increased the mean age. Age is an independent risk factor for arterial stiffness. Many epidemiological studies have demonstrated a significant relationship between age and arterial stiffness, and showed the baPWV increased with age [20–23]. Whether genetic factors influence baPWV values in various races are not clear, which needs to be investigated on genetic diversity and population structure.

Our study revealed that serum uric acid level is an important risk factor for arterial stiffness in the hypertension population. In the current study, we found that graded serum uric level does not have a uniform impact on arterial stiffness evaluated by baPWV, and high risk for arterial stiffness was observed in high serum uric level. This result was consistent with previous studies in general population [8, 9, 24]. Our study also demonstrated that the risk of serum uric acid on arterial stiffness differs in gender. The present finding suggested that the normal serum uric acid level plays a slight role for arterial stiffness in male, but its relative importance increases with the presence of hyperuricaemia. In contrast, serum uric acid is an important risk factor for arterial stiffness throughout the different graded serum uric acid levels in female, particularly when hyperuricaemia develops. Our results indicated that serum uric acid level plays a critical role in the progression of arterial stiffness in female than in male in the hypertension population. Previous studies have demonstrated that serum uric acid level does not have a uniform impact on both genders, it was significantly associated with increased aortic and peripheral arterial stiffness in males, while females with gout were at increased risk for acute myocardial infarction compared to males [25–28]. Moreover, it has been revealed that serum uric acid level is an independent predictor of worsening coronary endothelial function only in females [29]. Endothelial dysfunction and inflammatory role for serum uric acid in the hypertension population could be a hypothesis that can account for increased arterial stiffness [30–32]. Previous animal model studies have shown that serum uric acid first activates the renin-angiotension system, inhibits nitric oxide, and leads to systemic vascular resistance, followed by a uric acid- mediated vasculopathy, involving afferent arterioles, leading to delayed sodium sensitive hypertension [33]. In addition, Oxidative stress related to uric acid metabolism also plays a critical role in the pathogenesis of hypertension [34]. A cross-sectional and 5-year follow-up study showed that inflammatory gene polymorphisms were not associated with arterial stiffness, but large-scale prospective studies are warranted [35]. The explicit mechanism of serum uric acid in arterial stiffness and its gender- specific effects still remain to be determined.

Several limitations should be considered in the interpretation of our results. First, this study was designed as a cross-sectional study, so the results do not infer causality and need to be further confirmed in a prospective study. Second, the enrolled subjects of our study were hypertension patients, and the use of different antihypertensive drugs and dosage may confound our findings, thus further research needs to examine the effect of different antihypertensive drugs and other concomitant medication on the association between serum uric acid level and arterial stiffness in the hypertension population. Despite the previous limitations, there are several strong points of this study. In particular, our analyzed data were obtained from a large sample of hypertension population, and the sample size of 10450 participants in this study can guarantee reliable conclusions. Additionally, all studied patients were enrolled from wards in hospitals where they information were kept with electronic medical records, which ensure the integrity and authenticity of clinical data. Moreover, because there is a difference in the normal values of uric acid in gender, and the number of females were more than that of males in this study, thus, we analyzed data by sex separately to reduce their bias and to arrive at a more reliable conclusion.

In conclusion, this study demonstrates that serum uric acid level could be considered as an important risk factor for arterial stiffness both in gender in the hypertension population. Further studies are required that have a larger number of patients and follow-up study to define the value of serum uric acid level in arterial stiffness.

## MATERIALS AND METHODS

### Ethics

This study was performed according to the principles of the Declaration of Helsinki, and was approved by the ethics committee of the Changhai Hospital, Naval Military Medical University, Shanghai, China. We obtained informed consent from patients prior to sample collection.

### Design

This was a cross-sectional study, designed to explore the correlation between serum uric acid level and arterial stiffness in the hypertension population. Patients were recruited from the Changhai hospital, No.908 hospital, Baoshan and Chongming district community health service centers in China from January 2008 to June 2017. The data were collected by hospital interviews. Demographics, clinical profile and laboratory data were based on electronic medical records in hospital and health service centers to search for the key words hypertension, cholesterol, arterial stiffness, and serum uric acid to identify all hospitalized patients with hypertension.

### Study subjects

Included subjects had a diagnosis of hypertension according to WHO/ISH (1999) diagnostic standards [36]. The inclusion criteria were (1) a blood pressure of ≥140/90 mmHg in individuals not on antihypertensive therapy, or (2) previously diagnosed hypertension in individuals taking antihypertensive therapy, (3) use of the following one or more antihypertensive drugs: diuretics, alpha- and beta-blockers, calcium channel blockers (CCB), angiotensin converting enzyme inhibitor (ACEI) and angiotensin II receptor blockers (ARB), and (4) aged ≥41 years.

The exclusion criteria were (1) unreliable to the questionnaire, (2) unavailable to write documentation, and (3) data were not available for review, including unintegrated clinical and laboratory data.

### Clinical and laboratory data

All participants were administered a standardized questionnaire that provided information related to occupation, medical history, past and current medication use, and personal habits such as cigarette, alcohol consumption and dietary habit.

Fasting venous blood samples were collected on admission to measure serum uric acid, gamma-GTP, creatinine, fasting glucose, and lipids, including total cholesterol, HDL cholesterol, and triglycerides. Additionally, systolic blood pressure was taken using an automatic blood pressure cuff, and the value was obtained by averaging three consecutive blood pressure measurements. BMI was calculated from weight and height measurements.

### Measurement of baPWV

The baPWV was automatically measured using form PWV/ABI instruments (PWV/ABI, BP-203RPE; Omron-Colin, Japan) as previously described [37, 38] by trained volunteers from local medical colleges. The validations of this automatic device and its reproducibility have been previously published [6]. The measurements of baPWV on both sides of arm and ankle were averaged to obtain the mean baPWV value. High baPWV was defined as the highest quartile of the values among the study subjects [8].

### Statistical analysis

Categorical variables were presented as counts and percentages and were analyzed by Fisher’s exact tests or the chi-square tests. Continuous variables were reported as the means and standard deviations for data of normal distribution, which were analyzed by One-Way Anova analysis, and they were reported as medians and interquartile ranges for data of abnormal distribution, which were analyzed by Kruskal-Wallis tests. The association between serum uric acid and baPWV and high baPWV was assessed by linear curve fitting analysis and multiple logistic regression analysis, respectively. Both non-adjusted and multivariate adjusted models were applied, and interaction and stratified analyses were conducted. Serum uric acid level was log-transformed for analyses and evaluated by quartiles. High baPWV was evaluated as categorical variables divided by the highest quartile of the values. Statistical analyses were performed using Statistical Package of the Social Sciences Software version 21.0 (SPSS, Chicago, IL, USA), and statistical graphics were generated using GraphPad PRISM 6 (Graph Pad Software Inc., San Diego, CA, USA). The level of significance was set with a two-tailed *p*-value of <0.05.

## Supplementary Material

Supplementary Figure 1

Supplementary Tables
