# Supplementary material for: High serum uric acid is associated with increased arterial stiffness in hypertension
Source: Aging (Albany NY). 2020 Jul 23;12(14):14569–81. doi: 10.18632/aging.103506 (PMC7425441; doi:10.18632/aging.103506)
Supplement: Supplementary Figure 1 [file aging-12-103506-s001..pdf]

SUPPLEMENTARY FIGURE

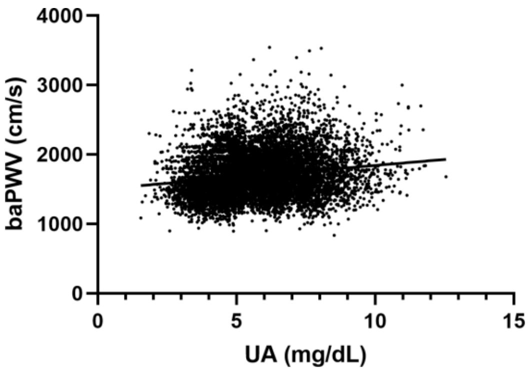

Supplementary Figure 1. Scatter diagram between serum uric acid level and baPWV. Scatter diagram showed a positive correlation between UA and baPWV in all patients.
