# Supplementary material for: High serum uric acid is associated with increased arterial stiffness in hypertension
Source: Aging (Albany NY). 2020 Jul 23;12(14):14569–81. doi: 10.18632/aging.103506 (PMC7425441; doi:10.18632/aging.103506)
Supplement: Supplementary Tables [file aging-12-103506-s002..pdf]

SUPPLEMENTARY TABLES

Supplementary Table 1. Univariate analysis of high baPWV.

Variable	Statistics	OR (95%CI)	*p* value
**Female**			
Age (year)	63.77+7.38	1.14 (1.12, 1.15)	<0.001
BMI (kg/m^2)	25.72+3.88	0.95 (0.93, 0.96)	<0.001
SBP (mmHg)	140.36+14.01	1.05 (1.04, 1.05)	<0.001
DBP (mmHg)	82.96+8.16	0.99 (0.99, 1.00)	0.110
Heart rate (bpm)	77.60+11.23	1.04 (1.03, 1.04)	<0.001
GGT (U/L)	20.94+18.03	1.01 (1.01, 1.01)	<0.001
Creatinine (μmol/L)	61.80+18.58	1.01 (1.00, 1.01)	<0.001
Fasting glucose (mmol/L)	6.28+1.95	1.10 (1.07, 1.13)	<0.001
Total-cholesterol (mg/dL)	5.43+1.11	1.14 (1.08, 1.20)	<0.001
HDL-cholesterol (mg/dL)	1.26+0.29	0.78 (0.63, 0.96)	0.017
Triglycerides (mg/dL)	1.98+1.50	1.09 (1.05, 1.14)	<0.001
Smoking			
No smoking	5353 (96.00%)	1.0	
Former smoking	88 (1.58%)	1.74 (1.12, 2.70)	0.013
Current smoking	135 (2.42%)	1.38 (0.95, 1.99)	0.088
Antihypertensive drugs	5171 (92.14%)	1.19 (0.93, 1.52)	0.165
**Male**			
Age (year)	65.79+7.38	1.00 (0.99, 1.01)	0.908
BMI (kg/m^2)	23.98+3.60	1.01 (0.99, 1.02)	0.517
SBP (mmHg)	139.98+12.68	1.00 (0.99, 1.01)	0.956
DBP (mmHg)	83.96+8.47	1.00 (1.00, 1.01)	0.374
Heart rate (bpm)	75.84+11.81	1.00 (0.99, 1.00)	0.753
GGT (U/L)	33.05+44.95	1.00 (1.00, 1.00)	<0.001
Creatinine (μmol/L)	80.62+32.05	1.00 (1.00, 1.01)	<0.001
Fasting glucose (mmol/L)	6.17+1.82	0.98 (0.94, 1.02)	0.306
Total-cholesterol (mg/dL)	5.02+1.03	1.00 (0.94, 1.07)	0.997
HDL-cholesterol (mg/dL)	1.28+0.33	0.99 (0.82, 1.21)	0.942
Triglycerides (mg/dL)	1.52+1.34	1.02 (0.98, 1.07)	0.314
Smoking			
No smoking	1210 (25.44%)	1.0	
Former smoking	1210 (25.44%)	0.95 (0.79, 1.15)	0.604
Current smoking	2337 (49.13%)	1.00 (0.85, 1.17)	0.977
Antihypertensive drugs	4478 (92.56%)	0.83 (0.63, 1.09)	0.175

Supplementary Table 2. Hierarchical analysis on relationship of UA and High baPWV.

Hierarchical factor		Male			Female		
		Events (n/patients)	OR (95%CI) *p*	*p* for interaction	Events (n/patients)	OR (95%CI) *p*	*p* for interaction
**Age**	**Age<65**	540/2157	1.11 (1.04, 1.19) 0.004	0.082	410/3190	1.25 (1.14, 1.37) <0.001	0.082
	**Age≥65**	675/2681	1.21 (1.13, 1.29) <0.001		994/2422	1.13 (1.05, 1.22) 0.001	
**BMI**	**BMI<25**	727/2953	1.20 (1.13, 1.28) <0.001	0.070	689/2449	1.12 (1.03, 1.22) 0.011	0.960
	**BMI≥25**	468/1811	1.10 (1.02, 1.19) 0.011		705/3123	1.12 (1.03, 1.21) 0.008	
**SBP**	**Q1**	290/1118	1.14 (1.04, 1.25) 0.006	0.600	136/1280	1.08 (0.95, 1.23) 0.253	0.660
	**Q2**	274/1117	1.11 (1.03, 1.24) 0.025		232/1288	1.13 (1.01, 1.27) 0.032	
	**Q3**	284/1119	1.19 (1.09, 1.31) <0.001		339/1283	1.19 (1.07, 1.32) 0.001	
	**Q4**	276/1119	1.21 (1.10, 1.33) <0.001		539/1286	1.11 (1.02, 1.22) 0.022	
**Creatinine**	**Q1**	268/1192	1.17 (1.06, 1.30) 0.003	0.002	307/1390	1.19 (1.04, 1.37) 0.013	0.055
	**Q2**	283/1200	1.03 (0.94, 1.14) 0.486		340/1372	1.13 (0.98, 1.30) 0.086	
	**Q3**	298/1194	1.12 (1.03, 1.23) 0.012		295/1400	1.43 (1.24, 1.65) <0.001	
	**Q4**	350/1203	1.33 (1.21, 1.46) <0.001		451/1402	1.14 (1.03, 1.26) 0.014	
**Smoke**	**Never**	306/1210	1.20 (1.09, 1.31) <0.001	0.398	1321/5353	1.14 (1.07, 1.21) <0.001	0.933
	**Former**	295/1210	1.21 (1.10, 1.33) <0.001		32/88	1.15 (0.68, 1.94) 0.612	
	**Current**	590/2337	1.13 (1.05, 1.21) <0.001		42/135	1.23 (0.82, 1.84) 0.322	
**Antihypertensive drugs**	**No**	77/271	1.31 (1.04, 1.64) 0.022	0.317	137/427	1.39 (1.08, 1.78) 0.010	0.097
	**Yes**	1108/4478	1.16 (1.11, 1.22) <0.001		1137/4762	1.12 (1.06, 1.20) <0.001	

Each stratification adjusted for all the factors (age, BMI, systolic blood pressure, diastolic blood pressure, heart rate, gamma-GTP, creatinine, fasting glucose, total cholesterol, HDL-cholesterol, triglycerides, smoking status, and antihypertensive drugs) except the stratification factor itself.
